# Enablers, barriers and strategies to build resilience among cancer survivors: a qualitative study protocol

**DOI:** 10.3389/fpsyg.2023.1049403

**Published:** 2023-07-18

**Authors:** Júlio Belo Fernandes, Josefa Domingos, Ana Silva Almeida, Cidália Castro, Aida Simões, Sónia Fernandes, Diana Vareta, Catarina Bernardes, Jorge Fonseca, Célia Vaz, Ana Rita Dias, Tatiana Fernandes, Catarina Godinho

**Affiliations:** ^1^Egas Moniz Center for Interdisciplinary Research (CiiEM), Egas Moniz School of Health and Science, Almada, Portugal; ^2^Nurs* Lab, Almada, Portugal; ^3^Department of Nursing, Centro Hospitalar de Setúbal EPE, Setúbal, Portugal; ^4^Department of Gastroenterology, Hospital Garcia de Orta EPE (HGO), Almada, Portugal; ^5^Department of Nursing, Centro Hospitalar Barreiro Montijo EPE, Barreiro, Portugal; ^6^Department of Nursing, Centro Hospitalar Universitário de Lisboa Central, Hospital Santo António dos Capuchos, Lisboa, Portugal

**Keywords:** cancer survivors, resilience, psychological, coping, challenges, facilitators, strategies

## Abstract

Cancer is a life-threatening illness affecting all dimensions of a person’s health. Cancer survivors must build resilience to face this adversity and continue their life projects. The present study explores the enablers, barriers, and strategies to build resilience among cancer survivors. This qualitative, descriptive exploratory study will use purposive sampling to recruit cancer survivors and healthcare professionals from two hospital centers in Lisbon and Tagus Valley. Interviews will be conducted until data saturation occurs. Data analysis will be performed using an inductive content analysis process with the help of the QDA Miner Lite database. The findings from this study will generate knowledge that may help stakeholders to identify effective strategies to build resilience among cancer survivors. By implementing strategies to foster resilience, healthcare professionals can potentially promote positive adaptations to cancer by strengthening resilience enablers and reducing the impact of barriers.

## 1. Introduction

Cancer is the second leading cause of death worldwide, responsible for more than 9.6 million deaths in 2018 (WHO, 2022). In 2020, there were 19.3 million newly diagnosed cancer cases worldwide (Sung et al., 2021). According to the data from the Global Cancer Observatory, Portugal recorded 60,467 new cancer cases and 30,168 deaths in 2020, with a 5-year prevalence rate of 1662.8 per 100,000 population (Global Cancer Observatory, 2021).

Cancer is a devastating disease that significantly impacts a person’s physical, emotional, and social well-being. The journey of cancer survivors is often filled with challenges such as physical side effects of treatment, emotional distress, financial burdens, and disruptions in daily life and relationships. However, many cancer survivors exhibit remarkable resilience and maintain a positive outlook despite this adversity. Building resilience is crucial for cancer survivors to continue pursuing their life goals and aspirations. Resilience is a concept that has gained increasing attention in the healthcare field. It refers to the ability to withstand adversity and bounce back from demanding and stressful life experiences (Wiig et al., 2020). Resilience involves upholding a stable path of healthy functioning over time, along with the ability to generate positive emotions and experiences despite challenging life events (WHO, 2017). Authors argue that resilience is not a specific characteristic but rather a multidimensional concept that implies experiencing a substantial threat or severe adversity and the ability to adapt effectively. Resilience allows the person to enhance their internal and external resources (Molina et al., 2014; Deshields et al., 2016) through a process that leads to developing a psychic construction suitable for social insertion despite the potential aggression (Luthar et al., 2000; Rutten et al., 2013).

There are numerous definitions of resilience (WHO, 2017). Despite their differences, the countless definitions indicate that resilience is linked to processes and skills that lead to positive individual and community health outcomes, notwithstanding serious threats and hazards (Bartley, 2006; WHO, 2017).

Several scales have been developed for measuring resilience. However, theoretic resilience frameworks are not related to attempts to measure resilience due to the inherit challenge of measuring the person’s adaptive capacity (Sturgess, 2016). Several authors argue that the different scales only measure characteristics of resilience and that it is possible to score well in some areas while scoring poorly in others (Connor and Davidson, 2003; Bonanno et al., 2011). When measuring resilience, individuals answer inquiries regarding their own experiences and perceptions. This assessment requires an awareness action (Sisto et al., 2019) where they need to identify their resources. Furthermore, other studies have associated resilience with personal characteristics such as self-efficacy, self-esteem, and optimism (Edward et al., 2019; Gao et al., 2019; Lee et al., 2019). There is also evidence that resilience is a process; therefore, a person can mobilize inner resources to achieve positive outcomes (Sisto et al., 2019; Stainton et al., 2019). There is also some consensus that resilience might be an intermediate rather than an outcome, demanding the mobilization of one’s inner resources to achieve positive well-being outcomes (Sturgess, 2016; Sisto et al., 2019; Stainton et al., 2019).

Researchers should consider resilience as a set of psychological phenomena that must be explored. These phenomena can be triggered and fade over the course of an individual’s life, manifesting in certain areas while being absent in others (Rutten et al., 2013).

Evidence supports the notion that resilience is not solely a fixed trait but rather a dynamic process that can be cultivated and developed through various interventions. This notion applies to individuals facing challenging circumstances, including cancer (Ludolph et al., 2019; Seiler and Jenewein, 2019; Üzar-Özçetin and Hiçdurmaz, 2019; Kim et al., 2021; Saleem et al., 2021; Laker et al., 2022; Sihvola et al., 2022; Vargas-Román et al., 2022). Moreover, cultivating resilience can enhance an individual’s ability to overcome future adversities (Ludolph et al., 2019; Seiler and Jenewein, 2019; Üzar-Özçetin and Hiçdurmaz, 2019). Several events directly linked to cancer can considerably disturb urvivors’ health and quality of life. Factors such as the type and stage of cancer, time of diagnosis, and prognosis of the disease can significantly disrupt their well-being (Sun et al., 2019; Festerling et al., 2022). Studies also show that the side effects of treatment changes in body image, social stigma, and the fear of recurrence also can disturb cancer survivors’ health and quality of life (Willems et al., 2016; Karimi et al., 2017; Hall et al., 2018; Melissant et al., 2019). There is also evidence that cancer survivors experience a high level of stress, anxiety, depression, and fear (Amigo Vázquez et al., 2011; Seib et al., 2018), which ultimately can lead them to a distressing experience that threatens their physical, mental, and emotional well-being (Silva et al., 2012). Furthermore, cancer patients may resort to negative coping strategies when confronted with emotionally challenging situations (Baghjari et al., 2017). Therefore it is vital to highlight that coping strategies can be taught through interventions (Lee et al., 2006). In this context, health professionals can play a crucial role by helping cancer survivors to build resilience. However, cancer survivors do not always receive the support they need (Perl et al., 2016). Interventions that foster resilience among cancer survivors have demonstrated positive outcomes in improving psychological well-being, enhancing coping mechanisms, and promoting adaptive responses to stress. A review of randomized controlled trials aiming to identify effective resilience-promoting interventions in cancer survivors has recognized the beneficial effects achieved by interventions based on positive psychology, supportive-expressive group therapy, behavioral therapy, or mindfulness, with considerable variation in individual effect sizes (Ludolph et al., 2019).

Psychosocial interventions, such as psychoeducation, counseling, and supportive care programs, are a valuable source of information, emotional support, and practical tools to navigate the challenges they encounter during their cancer journey. These interventions focus on enhancing patients’ knowledge about their illnesses, fostering effective coping skills, and promoting adaptive strategies for managing emotional distress (Ludolph et al., 2019; Seiler and Jenewein, 2019; Üzar-Özçetin and Hiçdurmaz, 2019; Kim et al., 2021; Saleem et al., 2021; Laker et al., 2022; Sihvola et al., 2022; Vargas-Román et al., 2022).

Evidence shows several factors that can act as enablers or barriers to resilience (Melillo and Ojeda, 2005; Fernandes et al., 2021a). A previous study among nursing personnel revealed that higher levels of concern about stigma were associated with lower levels of resilience (Hernandez et al., 2016). A prior study on caregivers of people with schizophrenia identified various barriers to family resilience, including lack of knowledge about the disease, social stigma, expressed emotion, involvement in the relationship, and blame (Melillo and Ojeda, 2005; Fernandes et al., 2021a). Furthermore, these factors might differ in various contexts, as the manifestation of these factors for one health domain does not influence others (Davydov et al., 2010).

There is a lack of studies exploring and understanding the enablers, barriers, and strategies involved in building resilience among cancer survivors. By gaining an understanding of these aspects, healthcare professionals can direct their efforts towards identifying solutions to address these factors and remove or reduce their impact. Hence, this study aims to explore the enablers, barriers, and strategies to resilience among cancer survivors using a qualitative design to gather further information about resilience to help healthcare professionals support this group of patients more effectively.

## 2. Materials and methods

### 2.1. Study design

Descriptive exploratory designs help summarize and understand an area of interest that has not been studied in depth in a specific context (Hunter et al., 2019; Doyle et al., 2020). The phenomena under study are the enablers, barriers, and strategies to resilience among cancer survivors. Considering the paucity of knowledge regarding this phenomenon, we will conduct a qualitative descriptive exploratory study.

We followed the consolidated criteria for reporting qualitative research (COREQ) checklist (Tong et al., 2007) to guarantee the quality of this study protocol.

### 2.2. Time period

**Figure 1:**
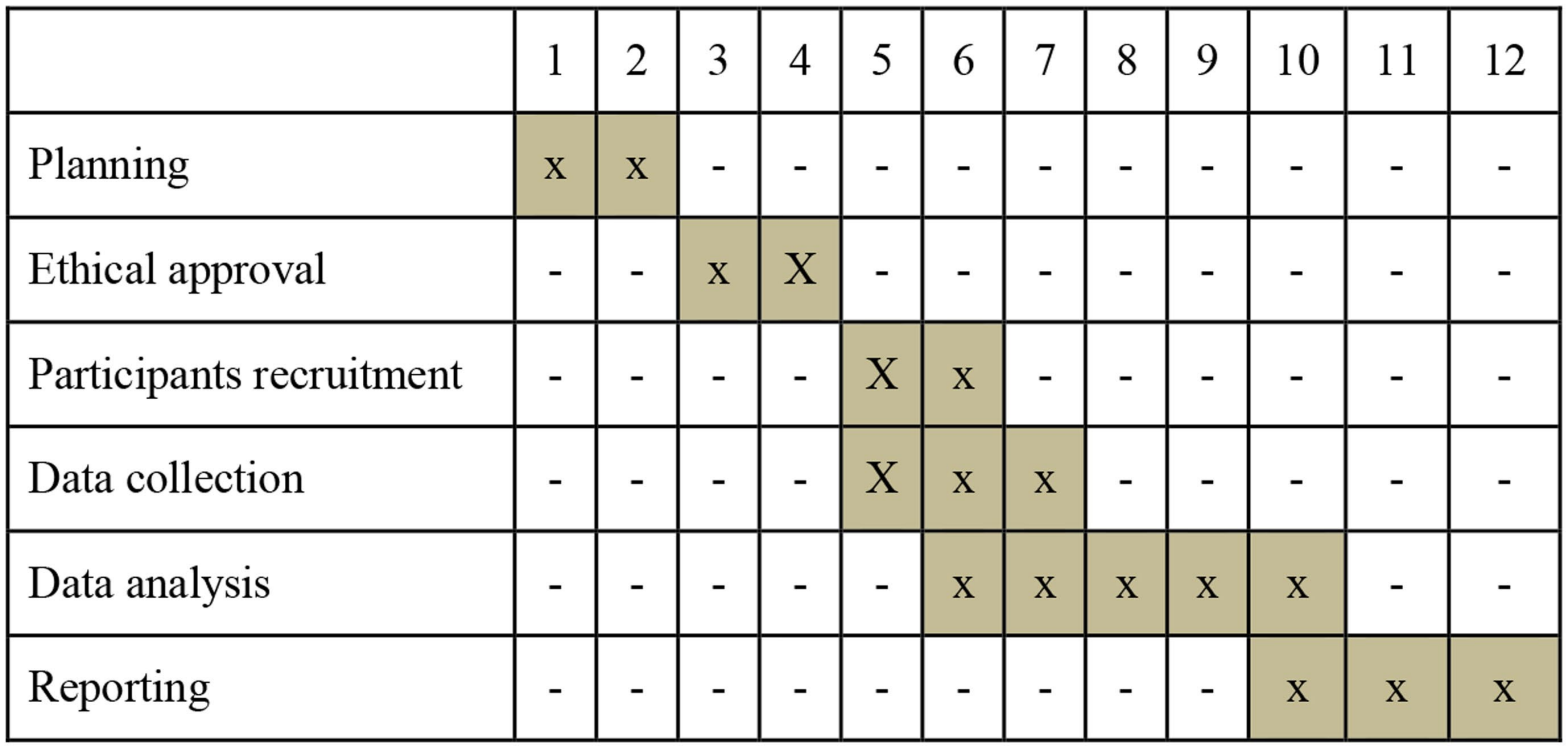
Project schedule.

September–October 2023–August 2024 (Gantt Chart Figure 1).

### 2.3. Population and recruitment

We will use purposive sampling to recruit participants from two hospital centers in Lisbon and Tagus Valley. This sampling method is widely used in qualitative research to identify and explore data related to the object of interest. It allows the researcher to collect data from the best-fit participants and consequently safeguard that the findings are pertinent to the research background (Palinkas et al., 2015).

We will select cancer survivors and healthcare professionals as participants to operationalize this research. By including cancer survivors as participants, we aim to capture their firsthand experiences and perceptions regarding resilience. These individuals have gone through the challenges of a cancer diagnosis, treatment, and the subsequent journey of survivorship. Their insights into the factors that have enabled their resilience, the barriers they have faced, and the strategies they have employed are invaluable for building knowledge regarding the phenomenon under study. Including healthcare professionals as participants is crucial as they play a significant role in supporting and caring for cancer survivors. Their expertise and experiences working with cancer survivors can shed light on the challenges patients face and the strategies they employ to foster resilience.

#### 2.3.1. Phase 1

The study population consists of cancer survivors.

For this investigation, we consider a cancer survivor as a person from the time of diagnosis until the end of life (National Cancer Institute, 2022).

#### 2.3.2. Phase 2

The study population comprises healthcare professionals (i.e., nurses, doctors, psychologists, and physiotherapists).

Researchers will purposefully select participants considering the cancer type, stage, and time of diagnosis (phase 1) and with different professions and ranges of time spent caregiving (phase 2) to include participants that will provide different lived experiences.

### 2.4. Inclusion criteria

#### 2.4.1. Phase 1

- Be a cancer survivor;
- People aged 18 years and above;
- Willingness to participate in the study;
- Ability to understand, provide informed consent and comply with all the proceedings.

#### 2.4.2. Phase 2

- Be a healthcare professional (i.e., nurses, doctors, psychologists, and physiotherapists);
- Previous experience in caring for cancer survivors;
- Pre-existing psychopathological disorder;
- Willingness to participate in the study.

### 2.5. Exclusion criteria

Target population under 18, and an unwillingness/inability to understand, provide informed consent or comply with all the proceedings.

### 2.6. Data collection procedures

We will contact eligible participants via telephone through the leading researcher who will present the research project, its aims, the concepts under study, and the importance of the participant’s collaboration. This will be followed by an email where we provide written information regarding the research project to allow participants to reflect on and analyze the concepts before deciding on participating in the study.

We will do the interviews individually and face-to-face in a private room at the hospital centers. Researchers will ensure the chosen location is noise-free in an environment that provides the participants privacy and comfort. No one else besides the interviewer and participants will be present during the interviews.

A nursing lecturer will conduct the interviews with a Ph.D. in Nursing Sciences, an experienced researcher and a skilled interviewer (Fernandes et al., 2021b, 2022a,c). The interviewer has no prior relationship with the participants.

Before the interview, the interviewer will remind the concepts under study and remind participants about the research aims and the importance of the participant’s collaboration, guaranteeing that all participants will sign the written informed consent. To capture the interview data accurately, each session will be digitally audio-recorded.

We will develop an interview guide to ensure discussion remains relevant to the study’s aims. The focus will consist of a set of open-ended questions to collect participants’ characteristics and perceptions, covering the following:

- Phase 1 - the participants’ characteristics (sex, age, marital status, time of diagnosis, type, and stage of cancer) and their perceptions regarding enablers, barriers, and strategies to build resilience;
- Phase 2 - the participants’ characteristics (sex, age, professional experience, time of expertise in caring for cancer survivors) and their perceptions regarding enablers, barriers, and strategies to build resilience.

We will use open-ended questions with prompts. For example, “What is your understanding of strategies you can use to build resilience among cancer survivors? Can you tell me more about that?”

We will pilot-test the interview guide among participants and experts in qualitative research to ensure it is understandable and helpful in retrieving the required information. In addition, we will collect data to determine that the guide is sufficiently explicit, objective, and comprehensive and does not present questions that could be ambiguous or equivocal.

We estimate that the interview will take approximately 40–50 min.

### 2.7. Data analysis

Audio-recorded interviews will be transcribed verbatim into a Microsoft Word® file and then returned to participants to consider any discrepancies and provide further clarification that may improve data accuracy.

The verbatim transcription will contemplate a unique code number (for example, P1, P2, P3, etc.) assigned to each participant to ensure the participants’ anonymity.

For the sample characterization, we will use the IBM Statistic Package for the Social Sciences software (IBM Corp. Released 2020. IBM SPSS Statistics for Windows, Version 27.0. Armonk, NY, USA: IBM Corp.) to perform descriptive statistic measures of the count, mean, standard deviation, median, minimum, and maximum.

The data analysis of open-ended questions will be executed concurrently with data collection. Two researchers will independently perform a conventional thematic analysis described by Braun, Clarke, Hayfield, and Terry (Braun et al., 2019) using the QDA Miner Lite database.

Researchers will repeatedly read and listen to the audio transcripts to derive concepts and themes. This step will allow findings to arise straight from the data analysis rather than from *a priori* expectations or models (Thomas, 2016), enabling the recognition of similarities and differences among the transcripts and the formation of patterns that allow the creation of initial themes. After, researchers will divide the transcriptions into different meaning units of words, phrases, and passages that emphasize on similar subjects and allocate codes to the meaning units and themes using the participants’ own words. The codes should reveal the similarities and differences in the participants’ perceptions concerning the subject in the study.

Researchers will discuss the broader initial codes and name themes and subthemes. If a consensus is not reached, a third researcher will analyze the discrepancy. Afterward, the entire research team will review the data analysis performed by the two researchers and match each quote to one of the identified themes. Lastly, the themes and organizing framework with other researchers external to the study and qualitative research experts will ratify the results.

### 2.8. Data saturation

For this study, we will focus on the richness of the selected cases than the sample size (Gupta et al., 2018; Vasileiou et al., 2018). Therefore, rather than setting a fixed sample size, we will consider saturation, as proposed by Glaser and Strauss (Glaser and Strauss, 2017), and carry on interviews until data has reached appropriate consistency to meet the study aims. The criteria to stop data collection is when data regarding a concept reveals no novel properties nor yields any further insights concerning the object of study.

### 2.9. Trustworthiness

Researchers will employ several procedures to ensure research rigor. Firstly, the sampling technique will allow researchers to make the most out of a small population of interest and arrive at valuable research outcomes (Palinkas et al., 2015; Moser and Korstjens, 2018). Therefore, purposive sampling will enable researchers to describe the phenomenon under study in all its nuances. Secondly, to guarantee data trustworthiness, the researchers will implement practices recommended by Nowell, Norris, White, and Moules (Nowell et al., 2017). To ensure credibility, the leading researcher will approach participants who met the study criteria to establish a good rapport and explain the study’s importance. Researchers will schedule face-to-face interviews with participants at times suitable for them and ensure each participant will be given enough time to share their feelings and experiences fully. During data analysis, researchers will discuss and detail every decision until consensus and return the initial themes and organizing framework to participants to ratify the researcher’s interpretations. Some cultural, social, and religious aspects can influence the participants’ perception regarding the phenomenon under study; therefore, to guarantee transferability, the participant’s responses will be followed where necessary during the interview to give comprehensive descriptions of their feelings and experiences. In addition, the final report will contain a detailed description of the participants’ characteristics and study setting along with the participant quotations to ensure that readers who sought to transfer the findings determine whether a transfer is feasible. Regarding the study’s dependability, researchers will provide the research process is logical and traceable; every step of the decision-making process will be detailed and documented. Lastly, to ensure confirmability, an external team of researcher experts in qualitative research will search for inconsistencies by comparing their perceptions with those of the researchers.

### 2.10. Ethics and procedures

Researchers will conduct the study according to the Declaration of Helsinki (revised in 2013). Therefore, the Hospital Centers’ Human Research Ethics Committee will review this study protocol. Furthermore, researchers will ensure all participants sign the informed consent form before the interview. Participants are free to not reply to some questions, change or review their answers, or voluntarily quit at any time. Each participant will be assigned a unique code number during data reporting to protect their anonymity and ensure confidentiality. No individual data will be accessible. Only the interviewer will have access to the identification sheet. Researchers will archive essential documents in a locked file, ensuring that they are readily available, upon request, to the competent authorities. The audio-recorded data will be destroyed after the verbatim transcription. All digital data will be coded and stored on a password-protected computer. All data will remain locked in a file cabinet at Egas Moniz University for 5 years. After this retention period, all data will be destroyed.

## 3. Discussion

Although researchers have expressed interest in resilience among cancer survivors for the last three decades (Edward et al., 2019; Ludolph et al., 2019), limited studies have explored and understood the enablers, barriers, and strategies to build resilience among cancer survivors. This research will provide an overview and a better understanding of key enablers, barriers, and strategies to resilience among cancer survivors by using qualitative research.

Resilience is a process that entails conscious action in addition to the identification and employment of personal resources (Sisto et al., 2019; Stainton et al., 2019). Its clinical relevance is well-known for patients with life-threatening diseases such as cancer. This life-threatening illness can profoundly impact the physical, mental, and emotional well-being of those diagnosed (Naughton and Weaver, 2014; Weitzel et al., 2022).

A growing body of literature has conclusively documented that cancer survivors can experience high stress, anxiety, depression, and fear (Amigo Vázquez et al., 2011; Seib et al., 2018). There is also evidence that, in some cases, cancer survivors live through it as a distressing experience that threatens their physical, mental, and emotional well-being (Silva et al., 2012). Cancer’s impact on them will vary according to factors that can enable or hinder resilience. In addition, some factors, like the spiritual, religious, cultural, and social context, may also act as enablers or barriers (Festerling et al., 2022; Yildirim Usenmez et al., 2022).

Deepening our understanding of the resilience process among cancer survivors is crucial. Studies show that resilience can be vital for developing post-traumatic growth (Dong et al., 2017; Zhang et al., 2017), a period through which patients develop skills that will benefit them in forthcoming situations.

Several studies have linked resilience with healthier adjustment to cancer, better mental health, increased quality of life, and treatment outcomes (Popa-Velea et al., 2017; Ye et al., 2017; Mohlin et al., 2021). In addition, building resilience in cancer survivors may yield better psychological adjustment and psychosocial functioning through cancer therapy (Seiler and Jenewein, 2019).

There is evidence that resilience-building strategies can be effective (Ludolph et al., 2019; Seiler and Jenewein, 2019; Üzar-Özçetin and Hiçdurmaz, 2019; Sihvola et al., 2022; Vargas-Román et al., 2022). For example, resilience training and stress management interventions can potentially strengthen psychological resilience in cancer survivors (Loprinzi et al., 2011). In addition, the strategies to promote resilience can contemplate different interventions by healthcare professionals and close relatives (Sihvola et al., 2022).

By developing strategies to foster resilience among cancer survivors, healthcare professionals will help them to emerge even stronger from this challenging life event, improving their coping strategies and adaptation in the face of adversity.

Like previously published study protocols (Chenevert et al., 2022; Garcia-Torres et al., 2022; Fernandes et al., 2022b), this study protocol will present the aims, methodological approach, and plan to operationalize the research.

This study has the potential to identify preexisting strategies, as well as enablers and barriers to resilience among cancer survivors. The findings can have practical implications for cancer survivors, healthcare professionals, and policymakers.

Understanding the enablers of resilience can help healthcare professionals tailor their interventions and support strategies to meet the unique needs of cancer survivors better. Incorporating an effective resilience-building strategy into clinical practice can enhance cancer survivors’ psychological well-being, coping mechanisms, and overall quality of life. In addition, the study findings can inform the development and enhancement of support programs for cancer survivors. Identifying the barriers to resilience can guide creating targeted interventions and resources that address these specific challenges. By implementing evidence-based strategies, support programs can better assist cancer survivors in developing the necessary skills, support networks, and psychological resilience to navigate the various challenges they may face throughout their journey.

The insights gained from the study findings can also contribute to formulating policies to support cancer survivors in building resilience. Policymakers can utilize the results to prioritize funding and resources for initiatives focusing on resilience-building programs, survivorship care planning, and psychosocial support services. Governments and healthcare organizations can foster a more comprehensive and patient-centered approach to cancer care by integrating resilience-promoting strategies into policy frameworks.

Finally, this study protocol can provide a solid foundation for future research in the field of psycho-oncology. It can inspire further investigations into the long-term effects of resilience on cancer survivorship outcomes, the effectiveness of specific interventions, and the role of social, cultural, and environmental factors in promoting resilience.

We identify some limitations to the study. Firstly, it’s important to acknowledge that different viewpoints may change over time and in different situations, so the findings may only apply to the specific context of this research. Second, by choosing to perform interviews, the participants’ perceptions and feelings might diverge from what they report due to a lack of confidence in ensuring anonymity or protecting identity, values, or beliefs. As detailed in this report, we have adopted several procedures to overcome potential limitations to ensure research rigor and compliance with the ethics procedures.

## 4. Conclusion

This study explores the enablers, barriers, and strategies to resilience among cancer survivors. To our knowledge, these particular concepts have not been thoroughly investigated.

The findings of this study have the potential to inform healthcare professionals, researchers, and policymakers in their efforts to promote resilience and improve the quality of life for cancer survivors.

## Author contributions

JFe, JD, AA, CC, AS, SF, DV, CB, JFo, and CG: conceptualization. JF and CG: supervision and project administration. All authors: methodology, writing—original draft preparation, and writing—review and editing. All authors have read and agreed to the published version of the manuscript.

## Conflict of interest

The authors declare that the research was conducted without any commercial or financial relationships that could be construed as a potential conflict of interest.

## Publisher’s note

All claims expressed in this article are solely those of the authors and do not necessarily represent those of their affiliated organizations, or those of the publisher, the editors and the reviewers. Any product that may be evaluated in this article, or claim that may be made by its manufacturer, is not guaranteed or endorsed by the publisher.
